# Incremental Learning with SVM for Multimodal Classification of Prostatic Adenocarcinoma

**DOI:** 10.1371/journal.pone.0093600

**Published:** 2014-04-03

**Authors:** José Fernando García Molina, Lei Zheng, Metin Sertdemir, Dietmar J. Dinter, Stefan Schönberg, Matthias Rädle

**Affiliations:** 1 Institute of Experimental Radiation Oncology, Department of Radiation Oncology, University Medical Center Mannheim, Heidelberg University, Mannheim, Germany; 2 Institute for Clinical Radiology and Nuclear Medicine, University Medical Center Mannheim, Heidelberg University, Mannheim, Germany; 3 Institute of Process Control and Innovative Energy Conversion (PI), Hochschule Mannheim, University of Applied Sciences, Mannheim, Germany; International Centre for Genetic Engineering and Biotechnology (ICGEB), India

## Abstract

Robust detection of prostatic cancer is a challenge due to the multitude of variants and their representation in MR images. We propose a pattern recognition system with an incremental learning ensemble algorithm using support vector machines (SVM) tackling this problem employing multimodal MR images and a texture-based information strategy. The proposed system integrates anatomic, texture, and functional features. The data set was preprocessed using B-Spline interpolation, bias field correction and intensity standardization. First- and second-order angular independent statistical approaches and rotation invariant local phase quantization (RI-LPQ) were utilized to quantify texture information. An incremental learning ensemble SVM was implemented to suit working conditions in medical applications and to improve effectiveness and robustness of the system. The probability estimation of cancer structures was calculated using SVM and the corresponding optimization was carried out with a heuristic method together with a 3-fold cross-validation methodology. We achieved an average sensitivity of 0.844±0.068 and a specificity of 0.780±0.038, which yielded superior or similar performance to current state of the art using a total database of only 41 slices from twelve patients with histological confirmed information, including cancerous, unhealthy non-cancerous and healthy prostate tissue. Our results show the feasibility of an ensemble SVM being able to learn additional information from new data while preserving previously acquired knowledge and preventing unlearning. The use of texture descriptors provides more salient discriminative patterns than the functional information used. Furthermore, the system improves selection of information, efficiency and robustness of the classification. The generated probability map enables radiologists to have a lower variability in diagnosis, decrease false negative rates and reduce the time to recognize and delineate structures in the prostate.

## Introduction

The most frequently diagnosed cancer for men in Germany, the UK and the US is prostatic adenocarcinoma [1]–[3]. New imaging techniques in MRI and their integration [4] have been developed with the aim of providing more information to radiologists, to improve visual interpretation and to formulate a better staging of prostate cancer. However, radiologists who perform clinical routines have to face a variety of MRI modalities, analyze a considerable amount of data and provide their evaluation. This routine requires substantial human interaction, and due to the challenging nature of interpreting and staging prostate cancer, data may be filtered cognitively or disregarded. Variations and inconsistencies in reports exist. MR imaging of the prostate has become so advanced that it presents a “dizzying array of data” for interpretation [5].

Various MR imaging modalities have been studied to improve visual inspection and diagnosis of cancer. MRI Diffusion-Weighted Imaging (DWI), MRI Dynamic-Contrast Enhanced (DCE) and MR-Spectroscopy [6]–[11] have been investigated. The general consensus is that combination of modalities increases the prediction accuracy of the diagnosis. However, the inter- and intra-variability in diagnosis and the significant amount of information to analyze have motivated the development of automated tools aimed at achieving a higher cancer detection rate and reducing clinical processing time simultaneously.

To reduce variability and inconsistency, automatic approaches for detection of prostate cancer using 1.5T multimodal imaging have been developed for *in vivo* studies on a per-voxel basis. The utility of multimodal imaging from coronal plane with 3D features (e.g. 3D co-occurrence matrix and a discrete cosine transform) and fisher linear discriminant (FLD) has been demonstrated using computer vision techniques [12]. The performance and robustness of second-order and first-order statistical discriminants alongside feature ensemble and random forest methods have also been enhanced [13], [14]. Statistical features and MR Spectroscopy have been used together with a multikernel graph embedding method demonstrating the contribution of multimodality classification [15]. The implemented second-order statistical approaches in these studies are based however on angular dependent features. Haralick [16] suggested a method invariant under rotation but the disadvantage is the computational cost involved, both in terms of memory requirement and computational time [17], [18]. We implemented an angular independent approach based on [17], integrating features with texture proprieties close to the human perception mechanism, which was also used by other medical applications [19]. We integrate features by considering the relationship between an element and all its neighboring elements at one time instead of one direction at a time, an approach based on [18]. In Madabhushi [13] a Gabor operator was employed to extract frequency signatures. However, the contribution of this method negatively affect the classification results. Other methods as local phase quantization (LPQ) [20] and local binary pattern (LBP) [21] have been utilized to quantify textural properties. Ojansivu [22] introduced a rotation invariant local phase quantization (RI-LPQ) to extract texture information. RI-LPQ outperformed Gabor operator as well as local binary patterns (LBP). Thus, RI-LPQ was implemented to identify frequency signatures.

Detection feasibility of cancer using support vector machines (SVM) and MR images (T2-weighted, apparent diffusion coefficient (ADC) derived from DWI and wash-in-rate (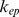) derived from DCE) has been demonstrated [23]–[25]. Moreover, the performance of SVM was enhanced in comparison with other classification methods for the recognition of prostate cancer using different MRI modalities (T2-weighted, DWI-ADC and pharmacokinetic parameters derived from DCE) [26], [27].

SVM performs well when the data set is limited in size and nonlinearly distributed. However, as is the case for any supervised classifier, performance and precision rely on the availability of a considerable data set size of correctly identified observations. Generally, the studies using SVM to learn from new data involves discarding the existing classifier, aggregating the new data to the old set and training a new classifier from scratch. As an effect, these methods result in unlearning [28], which is the inability of the system to learn new patterns without forgetting previously learned ones. Not forgetting old or underrepresented patterns is important for the recognition of prostate cancer because of the different possible features of cancer and its overlap with non-cancerous or normal structures. Such unlearning can be addressed through an incremental learning algorithm, defined as one that meets the following criteria [29], [30]:

1. be able to learn additional information from new data.
2. not require access to the original data used to train the existing classifier.
3. preserve previously acquired knowledge.

The main contribution of our framework is the implementation and evaluation of an incremental learning algorithm based on [30], which uses an ensemble algorithm inspired from Freund and Schapire’s adaptive boosting algorithm (Adaboost.M1) [31]. A random subspace method [32] was also implemented for the selection of features. Biological studies have shown that ensemble classifiers with SVM outperform single and balanced SVM [33]–[36]. Ensemble methods are one of the most promising solutions to many biological problems and together with SVM, as base classifier, further improvement in recognition of prostate cancer can be achieved. In this study we present a pattern recognition system to estimate the probability of prostate cancer using a multimodal data set from a real clinical environment.

## Materials and Methods

### Patient Data Set

The institutional review board (Institute for Clinical Radiology and Nuclear Medicine, University Medical Center Mannheim, Heidelberg University) approved this retrospective study. Patient information was anonymized and de-identified prior to any analysis. We used data of 12 patients from a clinical environment (full spectrum) with ages between 52 and 75 years, Gleason pattern of 3 and 4 with Gleason scores between 6 and 8. The data set contained in most cases other glandular non-cancerous prostate conditions like prostatitis, benign prostatic hyperplasia and prostatic atrophy.

The patients did not undergo any treatment before the acquisition of MR images. Treatments such as hormone therapy, previous biopsy or brachytherapy [12], [37], [38] tend to alter intensity values of the images, resulting in a drastically diminished ability to determine a threshold between cancer and benign intensity values.

### Acquisition of the MRI Data Set

The data set included MRI transversal slices with three modalities: T2-weighted, Dynamic-Contrast Enhanced plasma flow (DCE-PF) and DCE mean transit time (DCE-MTT). The three MRI modalities were acquired with a mean time of 45 minutes without interruption. This process aids to minimize tissue deformation and motion artifacts between the different MR images. A 3.0T scanner (Magnetom Trio; Siemens Healthcare, Erlangen, Germany) was used with a body phased array coil combined with an endorectal coil (Medrad Medical Systems, Volkach, Germany). The T2-weighted images were acquired with the following parameters: TR/TE = 4000/101 ms; Size of matrix = 320×320; echo-train length (ETL) = 25; Pixel bandwidth = 200 Hz; Field of view (FOV) = 20 cm; Pixel spacing = 0.625/0.625 mm; Slice thickness = 3 mm; Sequence type = TSE. The acquisition parameters for DCE were: TR/TE = 193/1.02 ms; Size of matrix = 384×388; Field of view (FOV) = 39 cm; Pixel spacing = 1.015/1.015 mm; Slice thickness = 6 mm; Sequence type = 2D TurboFlash T1; Flip angle = 9. The parametric maps, plasma flow and mean transit time were calculated using the open source perfusion analysis tool UMMPerfusion [39]. The DCE examination was performed after bolus injection of 0.1 mmol/kg body weight of gadolinium chelate (Dotarem; Guerbet, Roissy CdG Cedex, France) with a bolus velocity of 2.5 mL/s using a power injector (Medrad Inc, Pittsburgh, PA), followed by a saline flush of 40 mL.

### Histological Data Set

The group of patients had histologically confirmed cancer. The elapsed time between the acquisition of the MR images and the prostatectomy was less than 24 hours which minimizes tissue deformation artifacts, and low correlation between the pathological reports and the MR images due to anatomical movements [37], [40]. The histological report consists of different prostate cuts starting from the apex, middle and basis prostate region, separated in the right and left gland, serially sectioned according to a modified Stanford protocol [41]. A pathologist marked the corresponding cancer regions on each of the corresponding cuts.

### Description of the System

**Figure 1:**
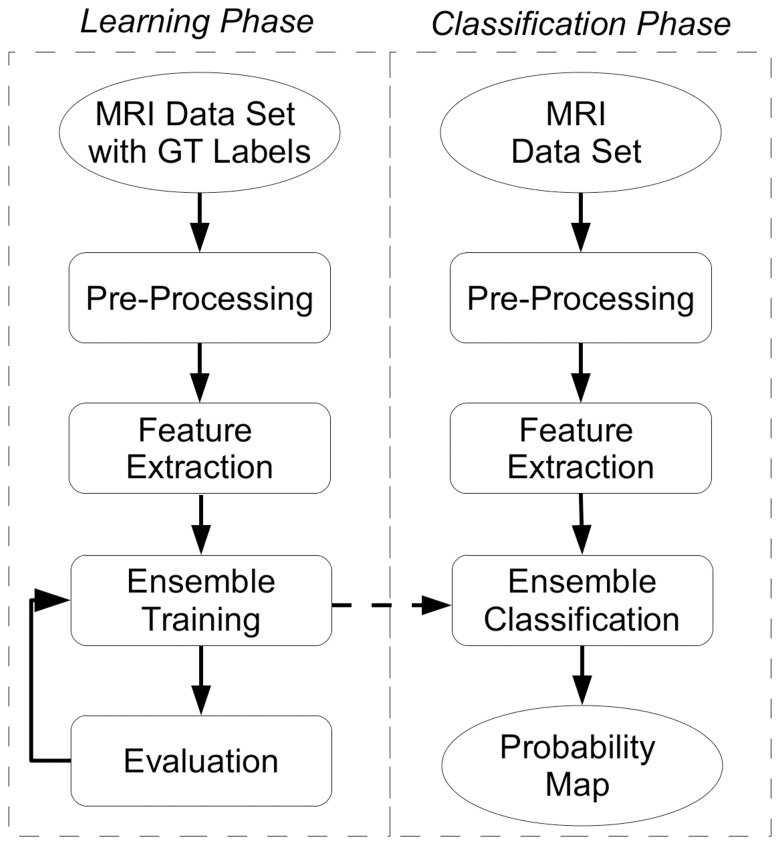
Framework Workflow. Sequence of the system components and overall organization for the estimation of prostate cancer.

This subsection outlines the components of the system. In Figure 1 the workflow of the pattern recognition system is shown. The two main phases of learning and classification are indicated in the figure. The pre-processing and feature extraction blocks were used in both phases. The datasets and the corresponding code are freely available upon request.

1. Ground Truth Labels: Labels of ground truth (GT) are necessary for the training and the evaluation of a supervised classifier. An experienced radiologist traced cancer regions on MR images, section by section, with the marked regions by a pathologist. A total database of 41 slices with about 70 cancerous ROIs from all twelve patients was used as ground truth.
2. Pre-Processing: Three steps were involved, a B-Spline interpolation for re-sampling of data, in which each pixel in different modalities corresponds to the same spatial location, a bias field correction using a variation of the nonparametric nonuniform intensity normalization algorithm (N3) and a landmark-based intensity standardization to minimize the intensity variation between slices.
3. Feature Extraction: Structural and functional information corresponding to T2-weighted, DCE-MTT and DCE-PF imaging was used, as well as first-order statistical descriptors (median, standard deviation, skewness, uniformity and average entropy), second-order descriptors using a neighboring gray level dependent matrix (NGLDM) and a neighborhood gray-tone difference matrix (NGTDM). To extract frequency signatures a rotation invariant local phase quantization (RI-LPQ) was implemented.
4. Training: The construction of a model to automate and formalize the pattern recognition process was implemented using a pixel-based supervised learning technique employing an ensemble SVM with the corresponding extension to the non-separable case [42].
5. Classification and Probability Map: The last step in the inductive inference process was the prediction/classification using the optimal model obtained after training. The posterior probability was also estimated to present the corresponding probability map.
6. Evaluation: The results were evaluated on accuracy, precision and efficiency as well as statistical significance. A methodology was also implemented to assess the results using area under the receiver operating characteristic curve (AUC-ROC) and to achieve an optimal generalized answer.

In the following subsections, we describe in detail the individual steps of our system.

### Ground Truth Labels

**Figure 2:**
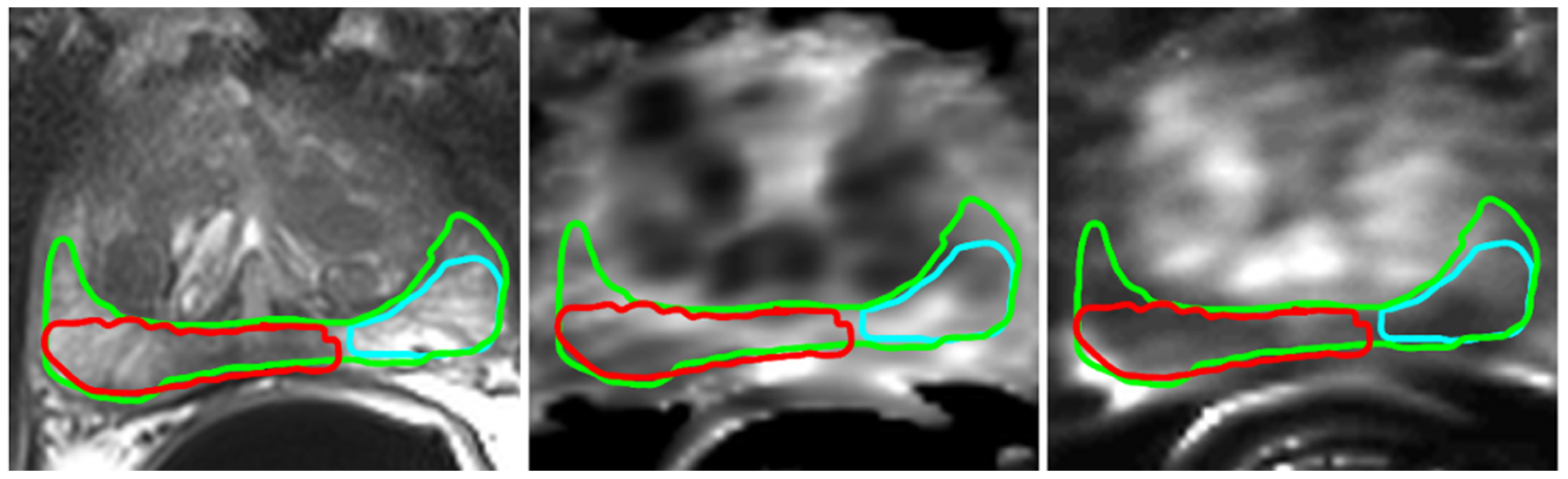
Ground Truth marks annotated over MRI modalities. Left a T2-weighted image, in the middle a DCE-MTT image and right a DCE-PF image are shown. The green line marks the peripheral zone (PZ), the red line marks the histologically confirmed cancer region and a sample of benign region is marked by a cyan line.

Ground truth (GT) labels for the learning phase was extracted using the annotations of an expert radiologist following the histological reports of a pathologist. The same process has been used in many investigations for the generation of GT [12], [23], [43] in cases where the conditions of clinical data are not suitable to perform an automatic registration. An expert radiologist can recognize landmarks such as morphology of central and peripheral zone (PZ) of the prostate, urethra, seminal vesicle, calcifications, etc. These landmarks help to recognize and trace benign and malignant regions in MRI. However, delineation still remains a difficult task for a human observer. Therefore, considering the recommendation of [44], a methodology for manual segmentation to minimize variation in the delineation process was followed (see Fig. 2):

1. Tracing: Regions of interest (ROI) were traced using Osirix software [45]. The ROIs were annotated on the T2-weighted images because of its high resolution and to facilitate anatomy recognition.
2. Magnification factor: We used a fixed factor (1∶800) for all data sets to annotate the ROIs.
3. Window/level value: The window width and center in the T2-weighted images were fixed depending on the minimal and maximal gray value in each volume.
4. Peripheral zone (PZ): Only areas inside the PZ were assessed. The radiologist performed the corresponding annotation of this zone on T2-weighted images. We gave special attention to the PZ not only to reduce high bias produced by non-cancerous prostate conditions such as benign prostatic hyperplasia located in the central zone [4], [46], [47], but also the PZ constitutes over 70% of the glandular prostate [46] and 65%–74% of the prostate tumor nodules are located in this zone [48].

After the annotations by the radiologist on T2-weighted images, the corresponding masked images constituting the ground truth were extracted. One precondition to minimize mislabeled pixels in the ground truth was the selection of a minimum size of cancer. Haider [40] pointed out that tumor sizes greater than 0.13 *cm* ^2^ are sufficient to detect significant cancer areas. Therefore sizes of cancer larger than 0.1 *cm* ^2^ were employed in this study.

### Pre-Processing

During image acquisition, an affine transformation matrix was recorded describing the spatial location (translation, direction and rotation) of each data set. Based on the transformation, a spatial re-sampling of the data sets DCE-MTT, DCE-PF was performed using ITK Toolkit [49] to obtain a new image in the same spatial space as the T2-weighted data. B-Spline interpolation was employed in the re-sampling to improve registration.

**Figure 3:**
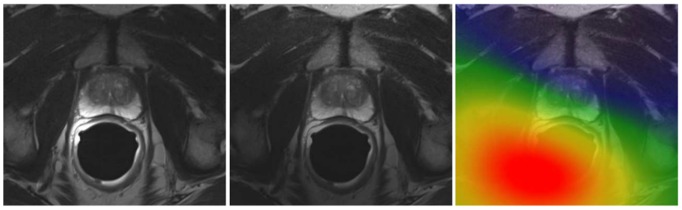
MRI Bias field correction. Left an uncorrected image, in the middle the corrected image and right image with the calculated bias field superimposed.

The PZ of the prostate suffers from a corruption of intensity values. To correct bias field artifact a variation of the popular nonparametric nonuniform intensity normalization (N3) algorithm [50], [51] was used. The algorithm, named “N4ITK”, connects a robust B-Spline algorithm with a multiresolution optimization which demonstrated a superior performance [52]. The parameters were set following the recommendation of [53]. Figure 3 illustrates an example of an image with the calculated bias field superimposed.

Another bias factor in MRI is non-standardness between slices. To overcome intensity variation a landmark-based method [54], [55] was applied because of its efficiency and accuracy [56]. The basic premise of a landmark-based method is to find a mapping based on the median and percentiles that deforms the histogram of an input image to match a reference. The mapping is performed linearly and independently per segments using three landmarks.

The landmark-based method consisted of two steps. First, a training step was executed only once for a given modality. The histogram 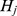 was computed for each slice 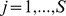 in a volume, where 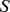 is the total number of slices. The median intensity (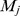) and the intensities corresponding to the 0 and 99.8 percentile (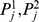) of 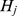 were computed (see [54] for details). After that, the median was mapped to the standard scale (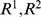) (equation 1).
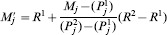
(1)


Finally, the mean value 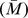 of mapped 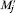 medians of each slice was computed.

The second step was the transformation of the intensity. In the transformation step, again the median intensity (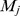) and intensities corresponding to the 0 and 99.8 percentile (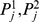) of 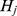 were calculated. The intensity values for each pixel 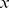 were mapped to a new value 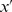:
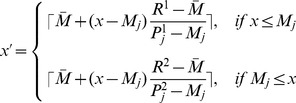
(2)


Finally, the set of images were normalized using z-scores such that intensity values have a mean of 0 and standard deviation of 1.

### Feature Extraction

A total of 32 features corresponding to structure (T2-weighted images (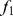) ), functional (DCE-MTT (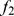), DCE-PF (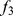) images) and statistical texture features were used to describe the appearance and shape of the intensity distributions of prostate structures. Texture information was extracted from the structural T2-weighted images.

Madabhushi [13] mentioned the importance of first- and second-order statistical features to improve the classification results. We consider some additional first-order descriptors of the intensity histogram based on [57]. We computed five operators with two windows sizes (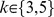) to get information on the median intensity (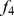), dispersion (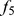), skewness (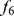), smoothness (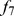) and variability (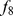) using the following operators respectively:

1. Median (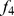)
2. Standard deviation (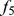)
3. Third moment (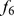)
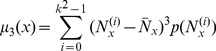
(3)
4. Energy (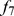)
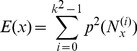
(4)
5. Entropy (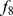)
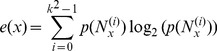
(5)

The operators were computed for each pixel 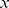 in all slices of a volume. 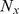 contains the 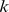-neighborhood pixels centered on 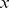 and 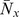 is the mean of 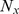. 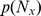 refers to the probability distribution of surrounding pixels.

Second-order statistical features were extracted using two angular independent approaches, a neighborhood gray-tone difference matrix (NGTDM) and a neighboring gray level dependent matrix (NGLDM).

In NGTDM, the matrix 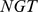 is set using the equation 6, 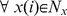 with 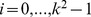.
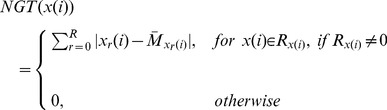
(6)


The array 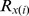 contains all pixels with gray value 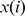, 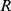 is the number of pixels in 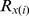. 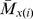 is the mean value over a neighborhood centered at, but excluding 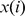. Five operators were computed with the NGT matrix (Coarseness (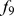), Contrast (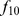), Busyness (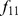), Complexity (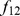) and Texture Strength (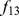)) [17]. These operators quantify the difference between regions with different intensity levels, the spatial frequency of intensity changes from one pixel to its neighbor and the magnitude of the difference between two intensity values, weighting the difference with their probabilities of occurrence.

For NGLM, equation 7 describes the implementation of matrix 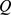. For a given image of size 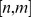 with pixel 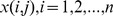 and 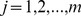, there is a matrix 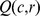, where c is the intensity value 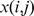 and 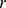 the number of repetitions of 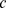.
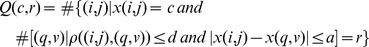
(7)


The neighborhood was defined by 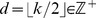, where 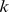 is the windows size (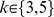). The points 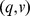 are the coordinates to localize the pixel 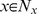. The symbol # denotes the number of elements in the set, 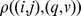 is the distance between the elements 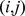 and 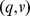, and 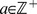 defines the similarity between two pixels (a = 10). Three operators were computed using the 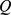 matrix (Number Nonuniformity (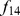), Second Moment (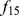) and Entropy (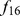)) [18].

To extract frequency signatures, a rotation invariant local phase quantization (RI-LPQ) implemented by Ojansivu [22] was integrated into the system (the Matlab implementation is available at the Outex site http://www.outex.oulu.). It is based on the blur invariance property of the Fourier phase spectrum. The phase information was extracted from a local k-neighborhood 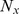 with k = 3, centered on a pixel x of a subimage I(x). The local spectra was computed with a short-term Fourier transform (STFT) defined by equation 8.
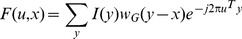
(8)


Where u is the frequency, and 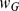 is a circular Gaussian function. A 2-D rotation matrix (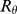) with an angle 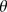 was used to rotate the images and the circular local neighborhood (36 different angles were generated). The local Fourier coefficients were computed and the phase information was recorded by observing the sign of the imaginary parts of the coefficients’ vector. The resulting values were normalized to a range of 0 to 15. A RI-LPQ histogram of these values with 16 bins was composed and used as 16 additional features (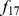 to 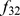) for each pixel in the classification.

### Training

The advantage of an incremental learning algorithm lies in the ability to incrementally learn additional information from new data when it becomes available. We implemented an ensemble algorithm based on [30]. The ensemble classifiers were trained based on a dynamically updated distribution over the training data points, in which points that are difficult to classify receive a higher probability to increase their chance of being selected into the next training data set. The algorithm uses a database 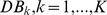 where 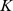 corresponds to the number of patients available (in our case 12 patients). The data points of all patients were first randomly permuted and split into K = 12 equal-sized parts.

As base classifier the non-separable case formulation of SVM was employed, which will be referred to 1C-SVM. The aim of training using 1C-SVM was to construct an optimal hypotheses 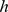, separating two classes and maximizing the distance to the closet point from either class [58], which is robust against model misspecification and provides a high predictive power. The main concept is finding an optimal hyperplane 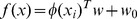 in the feature space using the formulation in equation (9).
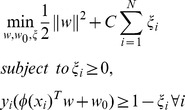
(9)where 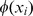 maps 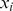 into a higher-dimentional space, 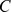 is a regularization hyper-parameter that penalizes misclassification. 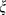 is a slack variable to relax the constraints and is called margin error or misclassification error.

The equation (9) can be solved with a quadratic programming solution using the lagrangian dual objective functions [59]. By using the dual objective function is possible to use the kernel trick 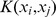, which maps data into higher dimensions in order to handle non-linear relationships between patterns. The mapping to a higher dimensionality also permits the capture of similarities between data points. A Gaussian Radial Basis Function (RBF) was used as a kernel, given by equation (10).
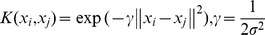
(10)



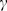
 is a hyper-parameter which changes the smoothness of the kernel function i.e. a greater or lesser relationship between the data points is found depending on the value of 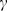.

To estimate the optimal hyper-parameter 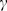 and 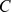 for 1C-SVM the heuristic method Nelder-Mead was used [60], [61]. The selected criteria to find the optimal classifier was the area under the receiver operating characteristic curve (AUC-ROC) which is commonly used in medical decision making, and is useful for organizing classifiers and used increasingly in machine learning and data mining [59], [62].

The inputs to the ensemble algorithm are:

1. training data 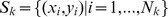. It consists of 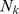 training data points with 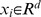 being the 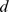-dimensional features and 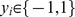 indicating the corresponding class. The 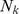 data points are randomly selected from the 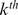 database 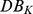;
2. a base classifier to generate a hypotheses 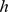. A requirement on the base classifier is that it obtains a 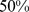 correct classification performance on its own training data set.
3. an integer 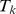 specifying the number of iterations 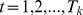 to be generated for each data set. 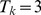 were employed, which was enough to reduce the prediction error.

The ensemble algorithm starts by initializing a set of weights 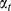 for the training data 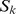, and a distribution 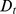 obtained from 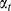. According to 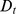 the training data 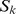 is divided into a training subset 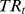 and a test subset 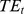 at the 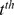 iteration of the algorithm. Without a priori information the distribution of weights 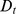 is initially set to be uniform. At each iteration 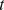, the weights adjusted at iteration 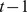 are divided by the sum of all 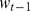 to ensure a legitimate distribution and a new 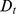 is obtained. Training and test subsets are drawn randomly according to 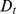, and the SVM is trained using tenfold CV method. A hypothesis 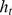 is obtained as the 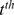 classifier, whose error 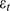 (11) is computed on the entire data set 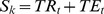.
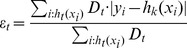
(11)


The error 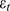 is required to be less than 0.5 to ensure a reasonable performance of 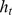. If the condition is satisfied, 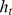 is accepted and the error is normalized to obtain the normalization error (12)
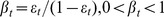
(12)


If the condition is not satisfied, then the current 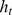 is discarded, and a new training subset is selected. All hypotheses generated so far are then combined using the weighted majority voting to obtain the composite hypothesis 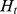 (13), which allows efficient incremental learning capability when new classes are introduced. The hypothesis with good performance are awarded a higher voting weight.
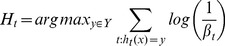
(13)


The error of the composite hypothesis 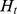 is computed with (14), and must also be less than 0.5 to ensure a reasonable performance of 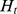, otherwise the algorithm discards the composite hypothesis and returns to select a new training subset.
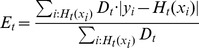
(14)


The normalized composite error is then computed with (15).
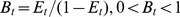
(15)


To reduce the weights of those data points that are correctly classified by the composite hypothesis 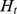 and lower the probability of being selected in the next training subset, the rule of equation 16 is used.
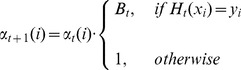
(16)


The hypothesis 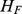 for the training subset and the subset of features can be obtained by combining all hypotheses that have been generated so far using the weighted majority voting rule (17).
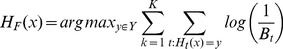
(17)


The features for use in the classifier was performed with a pairwise correlation using the spearman’s coefficients [63] avoiding multicollinearity. Features with a correlation coefficient lower than 0.60 were integrated in the pattern recognition system. Furthermore, a sequential forward methodology, leaving out the features that did not decrease significantly the prediction error, was employed.

Another method for the selection of features is a generalization of the random forest algorithm, known as random subspace ensemble [32], which perform better when the features have high correlation. A random subspace of incremental learning SVM for each training subset 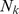 was implemented. The method randomly selected different subsets of features to train each classifier. Given the training subset 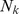, a subspace 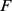 was chosen with 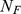 features. We employed 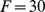 and 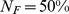 of the total number of features. All subspaces 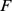 of incremental learning SVM were trained and combined using a weighted majority voting rule. To implement a random subspace the equation 17 was redefined as follow:
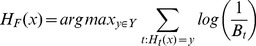
(18)


The normalized error 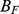 (19) was calculated with the mean square error (mse) of the hypothesis 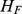. The final hypothesis 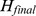 is computed with weighted majority voting (20).
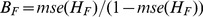
(19)

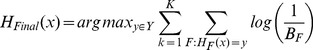
(20)


### Classification and Probability Map

The classification output of the SVM are decision values indicating the distance of data points to the hyper-plane without an estimation of how likely a data point belongs to a class, which is more important in a medical routine. We applied an extension of LIBSVM [42], which is an improvement of Platt [64], to give a probability estimation of cancer based on Lin et al. [65] and Wu et al. [66]. The extension proposes approximating the posterior probability by a sigmoid function using decision values and labels of each data point. Finally, the probability map estimation was saved in DICOM files for posterior analysis and diagnosis by the physicians.

### Evaluation

To achieve an objective evaluation, the following methodology was implemented. The data set was divided into three subsets: training, validation and test. The aim of the validation subset was to make a fair estimation of performance, independent of the test data, and to pick optimal parameters that maximize the performance of the validation subset which in turn provides a more generalized solution.

We implemented a nested cross-validation (CV) method for an unbiased estimation of prediction error in an independent test data. As used in [14], [15] threefold CV was used for the evaluation of the classifier, i.e. 8 patient studies were used for training and validation and 4 patient studies for independent testing. To estimate the unknown tuning parameters of the classifier tenfold CV was used with data points from the 8 studies selected for training and validation. The data points were randomly permuted and divided in 10 parts, one part for the validation and nine for training. The AUC of the receiver operating characteristic curve (ROC) was used as performance measure which provides a representation of the probability that a randomly chosen disease subject is correctly ranked with greater suspicion than a randomly chosen non-disease subject [67].

Since in other approaches other statistical measures of performance for binary classification were used, we also computed statistical measures, such as the sensitivity (TPR) and specificity (SPC) (21,22) as complementary information. To obtain binary classification a thresholding procedure is used. We selected a threshold 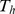 such that a data point 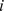 is classified as cancerous if the posterior probability 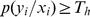, otherwise is classified as non-cancerous. Several threshold values 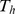 were selected and the balanced accuracy (BAC), which is defined as the mean of sensitivity and specificity, was computed for each of the threshold values. The selected 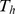 is the one which produced the maximum BAC value. The use of TPR and SPC yielded additional information about the performance of the classifier and allowed comparison of our results with other approaches. Additional measures like the Positive Predictive Value (PPV) (23) and the area under the PPV-TPR curve (AUC-PPV/TPR), known in machining learning as Precision-Recall curve, were also computed to give further information about performance on the positive class (cancer structure). PPV is also significant because it is also related with a priori chance of extra-capsular disease [37].
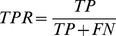
(21)

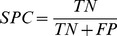
(22)

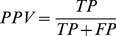
(23)TP and TN denote the number of true positive and true negative data points, respectively. FP and FN denote the number of false positive and false negative data points, respectively.

To evaluate the significance of the results between different models we used the Friedman test [68], which is a non-parametric statistical test that analyzes the ranked samples of an experiment and detects differences between the models across multiple test attempts. The p-value as a measure of statistical significance allows to reject or to accept the null hypothesis that there is no difference between experiments [69]. A p-value lower than 0.05 rejects the null hypothesis and the test is statistically significant, between 0.05 and 0.1 is marginal significant and higher than 0.1 is not statistically significant.

## Results

Feature generation, texture analysis, standardization, normalization, data cleaning, bootstrapping and evaluation methodology were implemented using Matlab 7.10 software. Training, prediction and the extensions of 1C-SVM were computed with LIBSVM C++ code [42]. To speed up the CV process and classification phase we implemented the code using the parallel computing toolbox of Matlab and the Grid Cluster infrastructure of Baden - Wuerttemberg bwGRID [70].

The results of accuracy, precision and efficiency are presented using the mean and standard deviation. We present also the corresponding box plots to give additional statistical information. The computational time reported was running the classifications on a PC with an Intel Core i7 CPU at 3.4 GHz and 16 GB of RAM.

### Performance of a Single 1C-SVM

A single 1C-SVM was run to compare the results with the proposed method. This classifier was implemented in other approaches for prostate classification giving good results [12], [23], [25], [26]. The features were selected according to the spearman’s coefficients and the sequential forward approach. The features that significantly improved the AUC-ROC were the first-order texture descriptors: standard deviation, third moment and energy. We compared three models using different configurations. The first model contained only values of T2-weighted images (T2), the second includes T2-weighted, DCE-MTT, DCE-PF (T2DCE) and the third model consisted of T2 and its most significant texture features (T2Tex). Table 1 shows the results of 1C-SVM using the three models.

**Table 1:** Classifier’s performance for a single 1C-SVM.

Measures ( 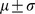 )	T2	T2DCE	T2Tex
TPR	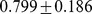	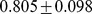	**0.811±0.094**
SPC	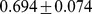	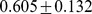	**0.739±0.091**
PPV	**0.311±0.171**	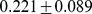	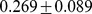
AUC-ROC	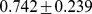	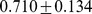	**0.813±0.080**
AUC-PPV/TPR	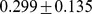	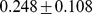	**0.376±0.101**
Time(min)	**27.31±7.65**	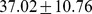	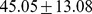

Three different combinations of features T2, T2DCE, T2Tex were assessed using the metrics TPR = Sensitivity, SPC = Specificity, PPV = Positive Predictive Value, AUC-ROC = Area under the receiver operating characteristic curve and AUC-PPV/TPR = Area under the Precision-Recall curve. The computational time given in minutes was the time required for the training and validation phase.

Mislabeled pixels or extreme intensity variations can be present in the training dataset. The identification of outliers in high-dimension for each class, without any assumption on the distribution, is challenging. To avoid deviant observation we used One-class SVM [42] in each class independently for each patient dataset. The parameters 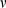 and 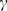, using a radial basis function, were optimized with Nelder-Mead. The training data set is subject to imbalance, known as minority class, that can cause class representation problems. Bootstrap over-sampling was used to balance the number of data points in each class for each patient. Bootstraping to enlarge the sampling pool [71] was performed with a Monte Carlo sampling algorithm [72]. The algorithm chose uniformly distributed pseudorandom data with replacement. To obtain a preferred sample size, the non-cancerous regions were undersampled, while the cancerous regions were oversampled until both classes were balanced. The algorithm was run for each patient independently. Another method to overcome the minority class problem is a cost-sensitive SVM [42] to penalize misclassification asymmetrically. However, we did not get a significant improvement compared to stratified sampling. For simplicity and reducing computing cost, we used the bootstrap sampling to balance the number of data points. Table 2 illustrates the results using a single 1C-SVM with a processed training dataset. Comparing table 1 and 2, the difficulties of a single SVM to handle outliers and an imbalanced data set can be observed. Including methods to avoid outliers and balance the data set contribute to enhance the performance of a single SVM. However, these procedures and a large number of samples introduced a significant computational cost during the training phase.

**Table 2:** Classifier’s performance for a single 1C-SVM with a processed training dataset.

Measures ( 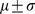 )	T2	T2DCE	T2Tex
TPR	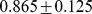	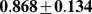	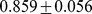
SPC	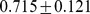	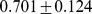	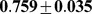
PPV	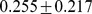	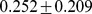	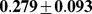
AUC-ROC	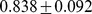	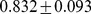	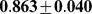
AUC-PPV/TPR	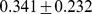	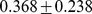	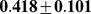
Time(min)	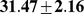	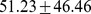	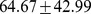

Three different combinations of features T2, T2DCE, T2Tex were assessed using the metrics TPR = Sensitivity, SPC = Specificity, PPV = Positive Predictive Value, AUC-ROC = Area under the receiver operating characteristic curve and AUC-PPV/TPR = Area under the Precision-Recall curve. The computational time given in minutes was the time required for the training and validation phase.

### Evaluation of the Incremental Learning Algorithm

**Figure 4:**
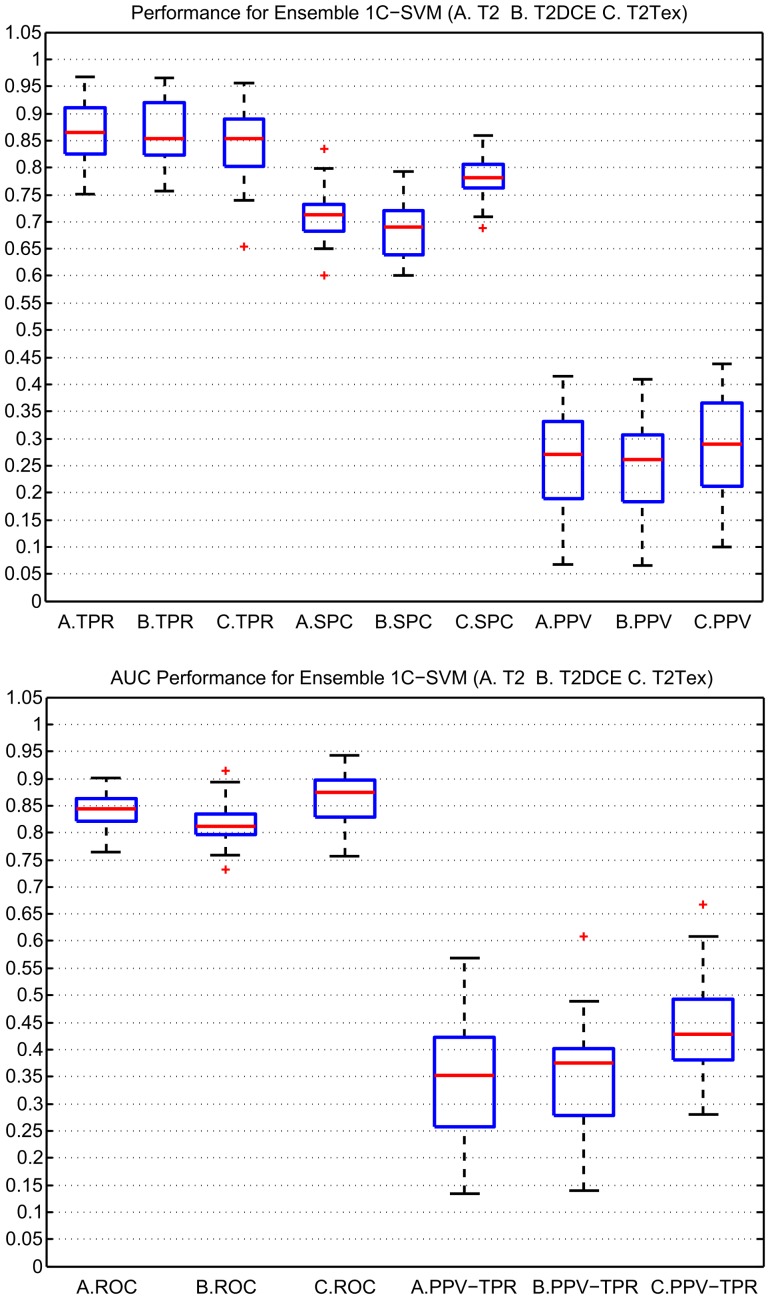
Classification results using an ensemble 1C-SVM. The performance of the three models (A. T2, B. T2DCE, C.T2Tex ) employing different metrics (TPR = Sensitivity, SPC = Specificity, PPV = Positive Predictive Value, AUC-ROC = Area under the receiver operating characteristic curve, AUC-PPV/TPR = Area under the Precision-Recall curve) is shown.

The same three models were compared (T2, T2DCE and T2Tex). Table 3 shows the results using the ensemble framework. The method based on Platt [42] to estimate the probability of cancer was implemented. We also used the decision values of SVM without the extension based on Platt to confirm the results. The AUC-ROC = 0.870±0.103 and AUC-PPV/TPR = 0.461±0.247 using the decision values did not present a significant difference. The model with T2-weighted and first-order texture descriptors (T2Tex) had the highest accuracy with an AUC-ROC significantly better than the T2 and T2DCE models (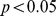), by highlighting relationship between pixels. Although T2 presented the highest sensitivity, T2Tex improved the trade-off between sensitivity, specificity and PPV values, which is more meaningful in clinical routines. T2DCE did not provide any significant improvement in performance. Figure 4 illustrates the box plot of these results. In comparison with table 2, the results of table 3 were not significantly different, however the ensemble 1C-SVM did not require a processed training data set, demonstrating that it is more robust in the presence of outliers and the imbalance in data than a single SVM. Furthermore, the computational cost in the training phase was a quarter of the time compared to a single SVM. A random subset of features was also evaluated, however we did not obtain a significant improvement (AUC-ROC = 0.848±0.083) and the computing time increased by a factor of 10.

**Table 3:** Classifier’s performance for an ensemble 1C-SVM.

Measures ( 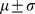 )	T2	T2DCE	T2Tex
TPR	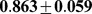	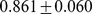	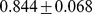
SPC	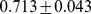	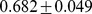	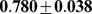
PPV	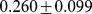	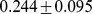	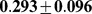
AUC-ROC	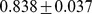	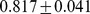	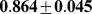
AUC-PPV/TPR	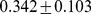	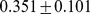	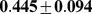
Time(min)	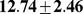	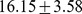	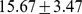

Three different combinations of features T2, T2DCE, T2Tex were assessed using the metrics TPR = Sensitivity, SPC = Specificity, PPV = Positive Predictive Value, AUC-ROC = Area under the receiver operating characteristic curve and AUC-PPV/TPR = Area under the Precision-Recall curve. The computational time given in minutes was the time required for the training and validation phase.

### Assessment of the Classifier According to the Area of Cancer Structures

Recognition of cancer in the PZ is challenging, due to the presence of other glandular non-cancerous prostate conditions, different stages and sizes of cancer. Therefore, we evaluated the performance of the framework using the two best models: T2 and T2Tex according to the size of cancer. Table 4 summarizes the results for two different sizes of cancer (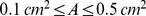 and 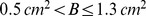). T2Tex performed better in AUC-ROC and AUC-PPV/TPR, for both small (A) and big areas (B), than T2, as well as a better trade-off between sensitivity, specificity and PPV. Texture information contributed also to identify suspect structures with different stages and sizes. In case of small areas (A) the false positive rate was higher and demonstrated discrimination complexity between small cancer areas and normal or other glandular non-cancerous prostate conditions. In case of big areas (B) the results were similar for both models.

**Table 4:** Classifier’s performance for different sizes of cancer structures.

	T2		T2Tex	
Measures ( 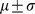 )	Region A	Region B	Region A	Region B
TPR	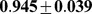	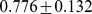	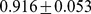	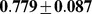
SPC	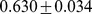	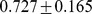	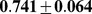	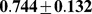
PPV	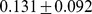	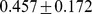	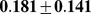	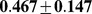
AUC-ROC	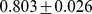	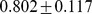	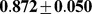	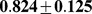
AUC-PPV/TPR	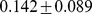	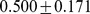	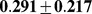	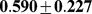

Two models were compared T2 and T2Tex using the metrics: TPR = Sensitivity, SPC = Specificity, PPV = Positive Predictive Value, AUC-ROC = Area under the receiver operating characteristic curve and AUC-PPV/TPR = Area under the Precision-Recall curve. Region A corresponds to cancer structures between 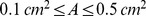 and region B corresponds to cancer structures between 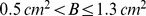. Five patients per region were evaluated.

### Evaluation of Ensemble 1C-SVM Against Human Experts

For comparison, two radiologists marked only cancer regions for five different patients (around 15 slices) in T2 images observing the usual MRI modalities and following normal evaluation routines (without any knowledge of the pathology or previous diagnosis). After the segmentation of cancer structures the results were evaluated against the ground truth. Similarly, the results of the automatic classification for the selected test patient were evaluated against the ground truth. Table 5 shows the comparison of cancer structures segmented by a radiologist and results obtained by our system. In terms of PPV, the radiologist can discern with more accuracy anatomical tissue or other non-cancerous prostate conditions that overlap with cancer structures. An expert who is familiarized with structures and shapes and prior knowledge about the meaning of other structures can further reduce the false positive rate. The sensitivity in the results by the human expert exemplifies the challenging nature of interpreting and staging prostate cancer. The human expert has to handle a variety of MRI modalities, MRI intensity variation artifacts and confined time for analysis. Our system helps to improve the sensitivity and decrease the time to recognize and delineate cancer regions.

**Table 5:** Evaluation of classifier against human experts.

Measures ( 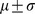 )	Observer 1	Observer 2	CAS
TPR	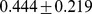	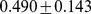	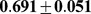
PPV	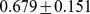	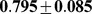	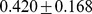
Time(sec)	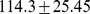	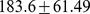	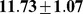

Five patients were evaluated by two radiologists and by the computer-aided system (CAS) using T2Tex. The performance was measured using TPR = Sensitivity and PPV = Positive Predictive Value. The time required to evaluate, recognize and delineate prostate cancer is given in seconds.

### Qualitative Evaluation

**Figure 5:**
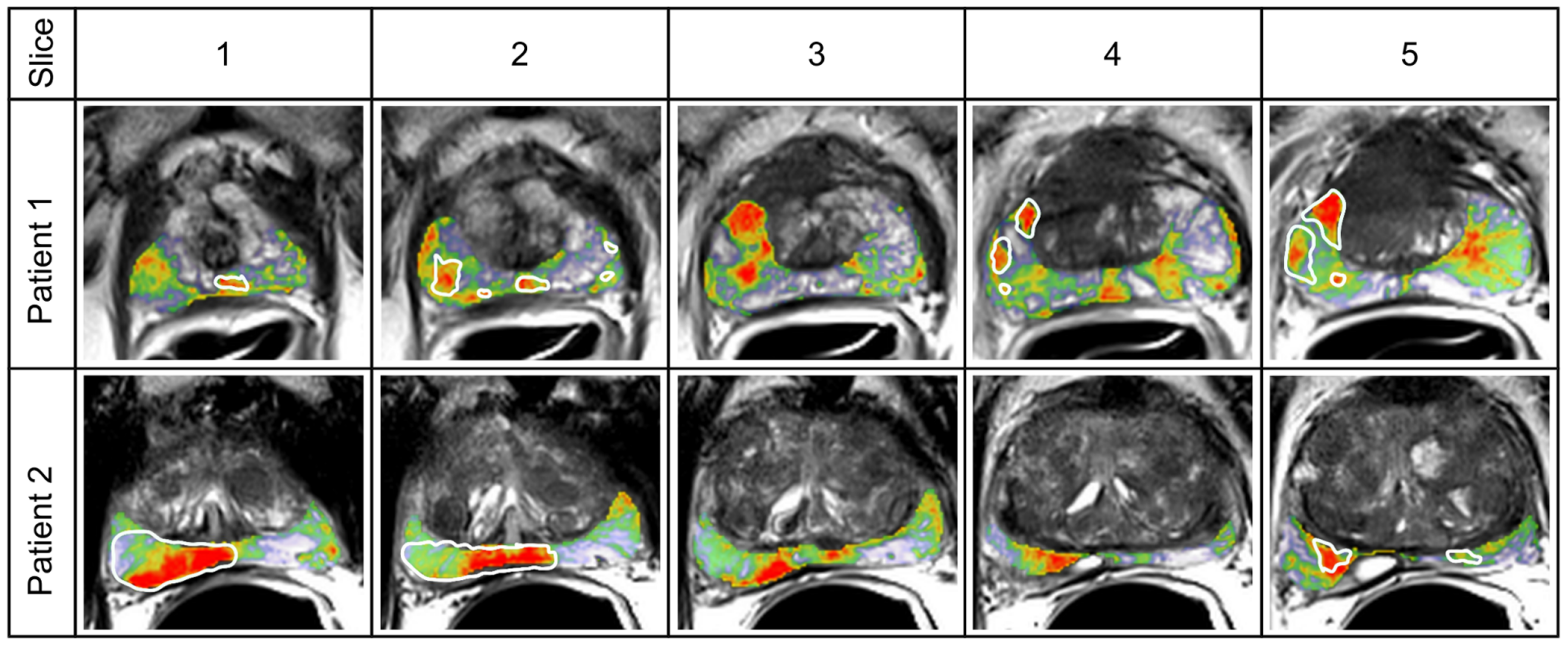
Recognition of cancer structures for two clinical patients. Five slices corresponding to the MRI volume of each patient are illustrated. The probability map was superimposed over the T2-weighted images. The background represents the gray values of the T2-weighted images for the whole prostate tissue. In the foreground the probability estimation of cancer is shown using a color map only over the PZ for the corresponding slices. The probability of cancer is ranged on a color scale: red (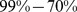 probability), yellow-green (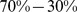 probability), without color (lower than 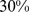 probability of cancer). The white marks highlight the ground truth regions.

Figure 5 illustrates the results after classification of two test patients using an ensemble 1C-SVM with T2Tex as features. A correlation between ground truth and classification results was observed on slices containing histological information. In neighbor slices was also observed evidence of cancer. These slices do not have histological data because of loss of information during the histology process for the extraction of prostate tissue. Nevertheless, the possibility of cancer in neighbor slices is evident due to the irregularities in shape and spread characteristics of cancer. The major number of T2-weighted slices together with the classification model contributes to locating suspicious cancer structures along the inter-slice direction.

In the presence of other non-cancerous prostate conditions, ensemble 1C-SVM classified these regions as suspicious tissue with a probability of cancer between 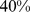 to 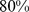, but the results depend on the stage of these prostatic conditions (e.g. first patient in the fifth slice on right side).

We trained the classifier with pixels inside the PZ. If regions outside the PZ or other prostate tissue (e.g ejaculatory ducts or neurovascular bundle) are classified, the results are meaningless. These regions have an overlap with cancer structures and were not included in the training.

Images such as the first and the second slice of patient 2 have to be assessed in detail with the radiologist and the pathologist to verify if this result was a statistical error of the classifier or a systematic error because of mislabeled data.

## Discussion

In this work we present the feasibility of developing an ensemble 1C-SVM. To the best of our knowledge, this is the first automatic classifier for recognition of prostate cancer that uses incremental learning algorithms satisfying the criteria of being able to learn additional information from new data while preserving previously acquired knowledge and preventing the unlearning [28] of old or underrepresented patterns. Moreover, the training phase does not need to be initiated from scratch when new data becomes available, actually it does not require access to the data used to train the existing classifier at all. This framework makes possible not only the integration of new discriminant features, but also accommodation of new classes that may be introduced with new data.

As mentioned in [25], [26], a comparison between study results is difficult because of differences between study population. In addition, each study has a different amount of data in which artifacts in the MR images and imbalances between cancer and benign data points are presented. Other differences involve annotations that do not follow same standards (e.g. classification of pixels, voxels, sextant), the discrimination task (e.g. using cancerous and normal tissue or normal tissue together with other non-cancerous conditions) and the evaluation methodology. We can report that with our framework an ensemble 1C-SVM renders a AUC-ROC of 0.864 (95% confidence interval(CI) = 0.847–0.880), sensitivity of 0.844 (95% CI = 0.819–0.868) and specificity of 0.780 (95% CI = 0.767–0.794), which is superior or similar to results reported in other studies [12], [14]. These studies were evaluated using leave-one-out (LOO), nevertheless it is known that LOO has a high variance [59]. Table 6 summarizes our results using both three-fold CV and LOO, as well as the results of current approaches. The dataset used for other studies is described as follows: Artan [25] used 21 slices from 21 patients. Niaf [26] experimented with 30 patients taking 42 cancerous ROIs. Tiwari [15] employed 29 patients and performed a classification per metavoxel.

**Table 6:** Summary of current studies and our system results.

Measures(%)	Artan	Niaf	Tiwari	OurFramework
TPR	64.0	–	–	84.40/84.69
SPC	78.0	–	–	78.03/78.62
PPV	–	–	–	29.29/29.38
AUC-ROC	79.0	89.0	85.0/89.0	86.38/86.85
AUC-PPV/TPR	–	–	–	44.53/46.69

The computer-aided studies from Artan [25], Niaf [26] used a leave-one-out (LOO) CV methodology, and from Tiwari [15] which, similar to this paper, used three-fold CV/LOO. Our results are reported for both methods three-fold CV/LOO. The metrics used are: TPR = Sensitivity, SPC = Specificity, PPV = Positive Predictive Value, AUC-ROC = Area under the receiver operating characteristic curve and AUC-PPV/TPR = Area under the Precision-Recall curve.

The analysis of cancerous structures is not trivial, they are characterized by their lack of shape and their overlap with other tissues (e.g. non-cancerous prostate conditions, and even healthy tissue). Moreover, the degree of intensity overlap depends also on the stage and size of malignant regions [9], [46] and together with the anatomy that change from patient to patient, makes the discrimination between benign and cancer tissue more difficult. Our results suggest that a more straightforward and efficient approach using first-order texture features is sufficient to achieve significant improvement in the recognition of cancer. Indeed, Niaf [26] demonstrated that using all extracted features would lead to poor performance. The degraded performance in our results using second-order features could reflect the differences in stage and size of malignant regions and the presence of other prostate conditions. However, other studies [13]–[15] suggest that second-order features yield good results. An evaluation of second-order angular independent features with a larger database containing more cancer regions (e.g. in different stages or different sizes) could help to assess the importance of these features in the classification. A random subspace of features did not improve the results of the incremental leaning algorithm. In Kuncheva [73] was reported a modest improvement using random subspaces depending on the data set. A restrictive choice of features and a higher number of random subspaces could help to increase the performance, but it implies higher computational cost, and we did not find evidence suggesting that a better choice of 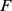 and 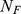 could improve the results significantly.

The data set was pre-processed to increase the performance of the classifier and robustness of the system. A general drawback of previous publications is the limited focus on pre-processing the data to minimize artifacts and deviations, e.g. the corruption of intensity values in the peripheral zone (PZ) of the prostate near to the rectum caused by sharp near field endorectal coil artifacts [12], [74] and the intensity variations between slices in MR imaging, known as non-standardness [54], [55], [75]. High signal intensity artifacts can not only limit the detection of cancer in the PZ, but also decrease the ability to generalize, which in most cases has not received appropriate attention in medical image applications. Although, these intensity variations have usually little impact on visual diagnosis, they significantly affect the performance of many image processing and analysis techniques based on absolute pixel intensities [76]–[78].

The ensemble workflow is similar to the decision process. A human being tends to seek several opinions before making any important decision. Individual opinions are weighted and combined to obtain a final decision [79]. This workflow offers higher accuracy and stability when compared to a single classifier. In addition, ensemble algorithms can often alleviate the problems of small sample size and high dimensionality which commonly occur in many bioinformatics applications [80].

The algorithm using ensemble 1C-SVM is also easy to implement and converges much faster than a single classifier because the ensemble algorithm does not use a large database for the training phase. Managing and analyzing huge databases implies computing complexity, storage problems and poor classification accuracy due to difficulties in finding correct classifiers [81], i.e. the size of the search space increases and underrepresented patterns that depend on the changing anatomy from patient to patient, may go undetected.

The incremental learning algorithm also demonstrates a more generalized performance and a narrow confidence interval, which indicates improved stability and robustness. Furthermore, this suits the working conditions in medical applications, in which a representative data set is difficult and time consuming to obtain. Data sets often become available in small and separate batches at different times.

The base classifier 1C-SVM method using the Gaussian Radial function also renders good results. The optimization process to find the hyper-parameters, together with the use of a radial kernel, was crucial to obtain higher accuracy. A Gaussian function can better characterize the similarities between cancer structures and neighbor pixels, which correspond in fact to the standard deviation of the intensity values. A default 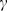 parameter depending on the number of features, as used in other studies, is not an optimal choice to find similarities between structures. A heuristic method, such as Nelder-Mead, is a better choice for model selection, however a poor starting point can lead to a local search. We suggest to first run a loose search to select the starting point, and then run Nelder-Mead for a precise search.

The results using areas of cancer confirmed the positive performance of our system for different sizes of cancer structures. In addition, we observed that in the presence of larger malignant structures, other regions such as the central zone or tissue outside the prostate capsule were also affected by cancer. This observation suggests that the PPV value is related to the chance of extra-capsular disease, as mentioned also in [37].

Our results demonstrated that inclusion of the functional features used in this study: DCE parameters, plasma flow (PF) and mean transit time (MTT) do not improve the classification results. However, Vos et al. [24] demonstrated the contribution of different DCE parameters, such as T1Static, Ve in the extravascular and extracellular space, transfer constant (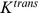) and rate constant (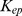). These DCE parameters have to be assessed with our system, which may improve the performance differentiating cancer and benign tissue. Other functional features such as Diffusion-Weighted Imaging (DWI) and MR-Spectroscopy have been evaluated [9]–[11], [15]. However, we do not integrate these features because of high bias and noise within the available data which are produced by artifacts in these modalities.

In our study, the ground truth was created manually by visual correlation of the histological sections with the MRI slices. There was not enough available information to perform an automatic registration, this is an important point to improve in the future. Nevertheless, visual correlation is still an acceptable method that has been used in other technical and clinical publications [12], [13], [15], [24], [26], [27].

Further investigations are necessary such as including more functional features in the system, increasing the number of patients and improving the extraction of ground truth. The integration of a third class corresponding to other non-cancerous prostate conditions is also important, for which the incremental learning algorithm can be improved e.g. using other combination rules or adding prior information in the ensemble to increase the performance.

## Conclusion

A pattern recognition system using an incremental learning algorithm and texture information for the automatic classification of prostatic adenocarcinoma, was presented. Our system not only suits the complex working conditions in medical applications, but also learns additional information from new data preventing unlearning. The performance, effectiveness and robustness of the system, along with its simplicity and speed in training as well in classification was also presented. Moreover, our system operates with a low number of features (T2-Weighted and texture operators) demonstrating the importance in the selection of information.

The probability estimation map generated by our system enables radiologist to diagnose with less variability in the diagnosis and reduce not only the false negative rate but also the time to recognize and delineate structures in the prostate. The radiologist could evaluate this estimation map before issuing a final report in order to suggest a better targeted treatment. As a result the radiologist can opt for a less aggressive therapy, can reduce the number of unnecessary biopsies and spare healthy tissue. In addition, our system can also be a suitable advisory tool for non-expert radiologists.

## References

[1] Society AC (2012) Cancer Facts & Figures 2012. Technical report, American Cancer Society.

[2] Cancer Research UK (2012) Prostate cancer - UK incidence statistics. Document, Cancer Research, UK. The latest UK prostate cancer incidence statistics from the Statistics team at Cancer Research UK.

[3] Institut RK (2012) Geschaetzte Zahl der Krebsneuerkrankungen in Deutschland nach Geschlecht 2008-Statistik. Technical report, Robert-Koch-Institut.

[4] Choi YJ, Kim JK, Kim N, Kim KW, Choi EK, et al (2007) Functional MR Imaging of Prostate Cancer. Radiographics 27: 63–75. 17234999 10.1148/rg.271065078

[5] Bassett M (2012). Group Advancing Prostate MRI Guidelines. Diagnostic Imaging.

[6] Luboldt W, Küfer R, Blumstein N, Toussaint T, Kluge A, et al (2008) Prostate Carcinoma: Diffusion-weighted Imaging as Potential Alternative to Conventional MR and 11C-Choline PET/CT for Detection of Bone Metastases. Radiology 249: 1017–1025. 18849502 10.1148/radiol.2492080038

[7] Kozlowski P, Chang S, Jones E, Berean K, Chen H, et al (2006) Combined diffusion-weighted and dynamic contrast-enhanced MRI for prostate cancer diagnosis–Correlation with biopsy and histopathology. Journal of Magnetic Resonance Imaging 24: 108–113. 16767709 10.1002/jmri.20626

[8] Alonzi R, Padhani AR, Allen C (2007) Dynamic contrast enhanced MRI in prostate cancer. European Journal of Radiology 63: 335–350. 17689907 10.1016/j.ejrad.2007.06.028

[9] Ocak I, Bernardo M, Metzger G, Barrett T, Pinto P, et al (2007) Dynamic Contrast-Enhanced MRI of Prostate Cancer at 3 T: A Study of Pharmacokinetic Parameters. American Journal of Roentgenology 189: W192–W201. 10.2214/AJR.06.1329 17885055

[10] Reinsberg S, Payne G, Riches S, Ashley S, Brewster J, et al (2007) Combined Use of Diffusion- Weighted MRI and 1H MR Spectroscopy to Increase Accuracy in Prostate Cancer Detection. ARRS 188: 91–98. 10.2214/AJR.05.2198 17179350

[11] Mazaheri Y, Shukla-Dave A, Hricak H, Fine SW, Zhang J, et al (2008) Prostate cancer: Identification with combined diffusion-weighted MR imaging and 3D 1H MR spectroscopic imagingcorrelation with pathologic findings. Radiology 246: 480–488. 18227542 10.1148/radiol.2462070368

[12] Chan I, Wells W, Mulkern R, Haker S, Zhang J, et al (2003) Detection of prostate cancer by integration of line-scan diffusion, T2-mapping and T2-weighted magnetic resonance imaging; a multichannel statistical classifier. Medical Physics 30: 2390–2398. 14528961 10.1118/1.1593633

[13] Madabhushi A, Feldman M, Metaxas D, Tomaszeweski J, Chute D (2005) Automated detection of prostatic adenocarcinoma from high-resolution ex vivo MRI. IEEE Transactions on Medical Imaging 24: 1611–1625. 16350920 10.1109/TMI.2005.859208

[14] Viswanath S, Bloch BN, Rosen M, Chappelow J, Rofsky N, et al. (2009) Integrating Structural and Functional Imaging for Computer Assisted Detection of Prostate Cancer on Multi-Protocol in vivo 3 Tesla MRI. SPIE Medical Imaging 7260. 10.1117/12.811899 PMC4188347 25301989

[15] Tiwari P, Kurhanewicz J, Madabhushi A (2013) Multi-kernel graph embedding for detection, gleason grading of prostate cancer via MRI/MRS. Medical Image Analysis 17: 219–235. 23294985 10.1016/j.media.2012.10.004 PMC3708492

[16] Haralick R, Shanmugam K, Dinstein I (1973) Textural features for image classification. IEEE Transactions on Systems, Man and Cybernetics 3: 610–621.

[17] Amadasun M, King R (1989) Textural features corresponding to textural properties. IEEE Transactions on Systems, Man and Cybernetics 19: 1264–1274.

[18] Sun C, Wee WG (1983) Neighboring gray level dependence matrix for texture classification. Computer Vision, Graphics, and Image Processing 23: 341–352.

[19] Yu H, Caldwell C, Mah K, Mozeg D (2009) Coregistered FDG PET/CT-Based textural characterization of head and neck cancer for radiation treatment planning. IEEE Transactions on Medical Imaging 28: 374–383. 19244009 10.1109/TMI.2008.2004425

[20] Ojansivu V, Heikkil J (2008) Blur insensitive texture classification using local phase quantization. In: Elmoataz A, Lezoray O, Nouboud F, Mammass D, editors, Image and Signal Processing, Springer Berlin Heidelberg, number 5099 in Lecture Notes in Computer Science. 236–243.

[21] Ojala T, Pietikäinen M, Mäenpää T (2002) Multiresolution gray-scale and rotation invariant texture classification with local binary patterns.

[22] Ojansivu V, Rahtu E, Heikkila J (2008) Rotation invariant local phase quantization for blur insensitive texture analysis. In: 19th International Conference on Pattern Recognition, 2008. ICPR 2008: 1–4.

[23] Vos P, Hambrock T, van de Hulsbergen-Kaa CA, Fuetterer JJ, Barentsz JO, et al (2008) Computerized analysis of prostate lesions in the peripheral zone using dynamic contrast enhanced MRI. Medical Physics 35: 888–899. 18404925 10.1118/1.2836419

[24] Vos P, Hambrock T, Barenstz JO, Huisman HJ (2010) Computer-assisted analysis of peripheral zone prostate lesions using T2-weighted and dynamic contrast enhanced T1-weighted MRI. Physics in medicine and biology 55: 1719–1734. 20197602 10.1088/0031-9155/55/6/012

[25] Artan Y, Haider MA, Langer DL, van der Kwast TH, Evans AJ, et al (2010) Prostate cancer localization with multispectral MRI using Cost-Sensitive support vector machines and conditional random fields. IEEE Transactions on Image Processing 19: 2444–2455. 20716496 10.1109/TIP.2010.2048612

[26] Niaf E, Rouviére O, Mége-Lechevallier F, Bratan F, Lartizien C (2012) Computer-aided diagnosis of prostate cancer in the peripheral zone using multiparametric MRI. Physics in medicine and biology 57: 3833–3851. 22640958 10.1088/0031-9155/57/12/3833

[27] Ozer S, Langer DL, Liu X, Haider MA, van der Kwast TH, et al (2010) Supervised and unsupervised methods for prostate cancer segmentation with multispectral MRI. Medical physics 37: 1873–1883. 20443509 10.1118/1.3359459

[28] French R (1999) Catastrophic forgetting in connectionist networks. Trends in cognitive sciences 3: 128–135. 10322466 10.1016/s1364-6613(99)01294-2

[29] Erdem Z, Polikar R, Gurgen F, Yumusak N (2005) Ensemble of SVMs for Incremental Learning. In: Oza NC, Polikar R, Kittler J, Roli F, editors, Multiple Classifier Systems, Springer Berlin Heidelberg, number 3541 in Lecture Notes in Computer Science. 246–256.

[30] Polikar R, Upda L, Upda S, Honavar V (2001) Learn++: an incremental learning algorithm for supervised neural networks. IEEE Transactions on Systems, Man, and Cybernetics, Part C: Applications and Reviews 31: 497–508.

[31] Freund Y, Schapire RE (1997) A decision-theoretic generalization of on-line learning and an application to boosting. Journal of Computer and System Sciences 55: 119–139.

[32] Ho TK (1998) The Random Subspace Method for Constructing Decision Forests. IEEE Trans Pattern Anal Mach Intell 20: 832–844.

[33] Kim HC, Pang S, Je HM, Kim D, Yang Bang S (2003) Constructing support vector machine ensemble. Pattern Recognition 36: 2757–2767.

[34] Peng Y (2006) A novel ensemble machine learning for robust microarray data classification. Computers in Biology and Medicine 36: 553–573. 15978569 10.1016/j.compbiomed.2005.04.001

[35] Caragea C, Sinapov J, Silvescu A, Dobbs D, Honavar V (2007) Glycosylation site prediction using ensembles of support vector machine classifiers. BMC Bioinformatics 8: 438. 17996106 10.1186/1471-2105-8-438 PMC2220009

[36] Guan Y, Myers CL, Hess DC, Barutcuoglu Z, Caudy AA, et al (2008) Predicting gene function in a hierarchical context with an ensemble of classifiers. Genome Biology 9: S3. 10.1186/gb-2008-9-s1-s3 PMC2447537 18613947

[37] Futterer J (2007) MR imaging in local staging of prostate cancer. European Journal of Radiology 63: 328–334. 17689908 10.1016/j.ejrad.2007.06.029

[38] Dinter D, Weidner A, Wenz F, Pelzer A, Michel M, et al (2010) Bildgebung der Prostata. Der Urologe 49: 963–975. 20628865 10.1007/s00120-010-2338-0

[39] Zoellner F, Weisser G, Reich M, Kaiser S, Schoenberg S, et al (2013) UMMPerfusion: an Open Source Software Tool Towards Quantitative MRI Perfusion Analysis in Clinical Routine. Journal of Digital Imaging 26(2): 344–352. 22832894 10.1007/s10278-012-9510-6 PMC3597952

[40] Haider MA, van der Kwast TH, Tanguay J, Evans AJ, Hashmi AT, et al (2007) Combined T2- Weighted and Diffusion-Weighted MRI for localization of prostate cancer. American Journal of Roentgenology 189: 323–328. 17646457 10.2214/AJR.07.2211

[41] Pelzer AE, Heinzelbecker J, Weiβ C, Frühbauer D, Weidner AM, et al (2012) Real-time sonoelastography compared to magnetic resonance imaging using four different modalities at 3.0T in the detection of prostate cancer: Strength and weaknesses. European journal of radiology 82(5): 814–821. 23273821 10.1016/j.ejrad.2012.11.035

[42] Chang CC, Lin CJ (2011) LIBSVM: A library for support vector machines. ACM Transactions on Intelligent Systems and Technology 2: 27 1–27: 27.

[43] Madabhushi A, Feldman M, Metaxas D, Chute D, Tomaszewski J (2003) A Novel Stochastic Combination of 3D Texture Features for Automated Segmentation of Prostatic Adenocarcinoma from High Resolution MRI. In: Lecture Notes in Computer Science, Berlin, Heidelberg: Springer Berlin Heidelberg. 581–591.

[44] Udupa JK, LeBlanc VR, Zhuge Y, Imielinska C, Schmidt H, et al (2006) A framework for evaluating image segmentation algorithms. Computerized Medical Imaging and Graphics 30: 75–87. 16584976 10.1016/j.compmedimag.2005.12.001

[45] Rosset A, Spadola L, Ratib O (2004) OsiriX: An Open-Source Software for Navigating in Multidimensional DICOM Images. Journal of Digital Imaging 17: 205–216. 15534753 10.1007/s10278-004-1014-6 PMC3046608

[46] Qi J, Shu-jie X (2012) Zonal differences in prostate diseases. Chinese Medical Journal 125: 1523–1528. 22800815

[47] McNeal JE (1981) The zonal anatomy of the prostate. The Prostate 2: 35–49. 7279811 10.1002/pros.2990020105

[48] Fuetterer J, Heijmink S, Scheenen T, Veltman J, Huisman HJ, et al (2006) Prostate Cancer Localization with Dynamic Contrast-enhanced MR Imaging and Proton MR Spectroscopic Imaging. Radiology 241: 449–458. 16966484 10.1148/radiol.2412051866

[49] Ibanez L, Schroeder W, Ng L, Cates J (2003) The ITK Software Guide: The Insight Segmentation and Registration Toolkit. Kitware Inc.

[50] Sled J, Zijdenbos A, Evans A (1998) A nonparametric method for automatic correction of intensity nonuniformity in MRI data. IEEE Transactions on Medical Imaging 17: 87–97. 9617910 10.1109/42.668698

[51] Arnold JB, Liow JS, Schaper KA, Stern JJ, Sled JG, et al (2001) Qualitative and quantitative evaluation of six algorithms for correcting intensity nonuniformity effects. NeuroImage 13: 931–943. 11304088 10.1006/nimg.2001.0756

[52] Tustison N, Avants B, Cook P, Zheng Y, Egan A, et al (2010) N4ITK:Improved N3 Bias Correction. IEEE Transactions on Medical Imaging 29: 1310–1320. 20378467 10.1109/TMI.2010.2046908 PMC3071855

[53] Tustison N, Gee J (2009) N4ITK: Nick’s N3 ITK Implementation For MRI Bias Field Correction. The Insight Journal.

[54] Madabhushi A, Udupa J (2005) Interplay between intensity standardization and inhomogeneity correction in MR image processing. IEEE Transactions on Medical Imaging 24: 561–576. 15889544 10.1109/TMI.2004.843256

[55] Nyúl LG, Udupa JK, Zhang X (2000) New variants of a method of MRI scale standardization. IEEE Transactions on Medical Imaging 19: 143–150. 10784285 10.1109/42.836373

[56] Bergeest JP, Jaeger F (2008) A comparison of five methods for signal intensity standardization in MRI. In: Bildverarbeitung für die Medizin 2008, Springer Berlin Heidelberg. 36–40.

[57] Gonzalez RC, Woods RE (2007) Digital Image Processing. Prentice Hall International, 3 edition.

[58] Vapnik VN (2000) The nature of statistical learning theory. New York: Springer, 2nd edition.

[59] Hastie T, Tibshirani R, Friedman JH (2003) The Elements of Statistical Learning. Springer.

[60] Nelder JA, Mead R (1965) A simplex method for function minimization. The Computer Journal 7: 308–313.

[61] de C Cosme R, Krohling RA (2011) Support vector machines applied to noisy data classification using differential evolution with local search. Technical report, Universidade Federal do Espirito Santo.

[62] Fawcett T (2006) An introduction to ROC analysis. Pattern Recognition Letters 27: 861–874.

[63] Gibbons JD, Chakraborti S (2003) Nonparametric Statistical Inference. CRC Press.

[64] Platt JC (2000) Probabilistic outputs for support vector machines and comparison to regularized likelihood methods. Advances in Large Margin Classifiers, Cambridge, MA.

[65] Lin HT, Lin CJ, Weng RC (2007) A note on platt’s probabilistic outputs for support vector machines. Technical Report 3, Journal Machine Learning, Hingham, MA, USA.

[66] Wu Tf, Lin CJ, Weng RC (2003) Probability estimates for multi-class classification by pairwise coupling. Journal of Machine Learning Research 5: 9751005.

[67] Hanley JA, McNeil BJ (1982) The meaning and use of the area under a receiver operating characteristic ROC curve. Radiology 143: 29–36. 7063747 10.1148/radiology.143.1.7063747

[68] Motulsky H (2009) Intuitive Biostatistics: A Nonmathematical Guide to Statistical Thinking. OUP USA, second edition.

[69] Ellison SL, Barwick VJ, Farrant TJD (2009) Practical Statistics for the Analytical Scientist: A Bench Guide. Royal Soc of Chemistry, 2 edition.

[70] bwGRiD (2012) member of the German D-Grid initiative, funded by the Ministry for Education and Research (Bundesministerium fuer Bildung und Forschung) and the Ministry for Science, Research and Arts Baden-Wuerttemberg (Ministerium fuer Wissenschaft, Forschung und Kunst Baden-Wuerttemberg).

[71] Namee BM, Cunningham P, Byrne S, Corrigan O (2002) The problem of bias in training data in regression problems in medical decision support. Artificial intelligence in medicine 24: 51–70. 11779685 10.1016/s0933-3657(01)00092-6

[72] Efron B, Tibshirani RJ (1994) An Introduction to the Bootstrap. Chapman and Hall/CRC, 1 edition.

[73] Kuncheva LI, Rodriguez JJ, Plumpton CO, Linden DEJ, Johnston SJ (2010) Random subspace ensembles for FMRI classification. IEEE transactions on medical imaging 29: 531–542. 20129853 10.1109/TMI.2009.2037756

[74] Liney GP, Turnbull LW, Knowles AJ (1998) A simple method for the correction of endorectal surface coil inhomogeneity in prostate imaging. Journal of Magnetic Resonance Imaging 8: 994–997. 9702904 10.1002/jmri.1880080432

[75] Simmons A, Tofts PS, Barker GJ, Arridge SR (1994) Sources of intensity non-uniformity in spin echo images at 1.5T. Magnetic Resonance in Medicine: Official Journal of the Society of Magnetic Resonance in Medicine/Society of Magnetic Resonance in Medicine 32: 121–128. 10.1002/mrm.1910320117 8084227

[76] Styner M, Brechbuhler C, Szckely G, Gerig G (2000) Parametric estimate of intensity inhomogeneities applied to MRI. IEEE Transactions on Medical Imaging 19: 153–165. 10875700 10.1109/42.845174

[77] Zhuge Y, Udupa JK (2009) Intensity Standardization Simplifies Brain MR Image Segmentation. Computer vision and image understanding: CVIU 113: 1095–1103. 20161360 10.1016/j.cviu.2009.06.003 PMC2777695

[78] Bagci U, Udupa J, Bai L (2010) The inuence of intensity standardization on medical image registration. Proc SPIE, Medical Imaging 2010: Visualization, Image-Guided Procedures, and Modeling 7625: 76251X–12.

[79] Polikar R (2006) Ensemble based systems in decision making. IEEE Circuits and Systems Magazine 6: 21–45.

[80] Yang P, Yang YH, Zhou BB, Zomaya AY (2010) A review of ensemble methods in bioinformatics. Current Bioinformatics 5: 296–308.

[81] Rokach L (2010) Ensemble-based classifiers. Artificial Intelligence Review 33: 1–39.

